# Beyond breast density: a review on the advancing role of parenchymal texture analysis in breast cancer risk assessment

**DOI:** 10.1186/s13058-016-0755-8

**Published:** 2016-09-20

**Authors:** Aimilia Gastounioti, Emily F. Conant, Despina Kontos

**Affiliations:** Department of Radiology, University of Pennsylvania, Philadelphia, PA 19104 USA

**Keywords:** Breast cancer risk, Parenchymal texture analysis, Quantitative breast imaging, Digital mammography

## Abstract

**Background:**

The assessment of a woman’s risk for developing breast cancer has become increasingly important for establishing personalized screening recommendations and forming preventive strategies. Studies have consistently shown a strong relationship between breast cancer risk and mammographic parenchymal patterns, typically assessed by percent mammographic density. This paper will review the advancing role of mammographic texture analysis as a potential novel approach to characterize the breast parenchymal tissue to augment conventional density assessment in breast cancer risk estimation.

**Main text:**

The analysis of mammographic texture provides refined, localized descriptors of parenchymal tissue complexity. Currently, there is growing evidence in support of textural features having the potential to augment the typically dichotomized descriptors (dense or not dense) of area or volumetric measures of breast density in breast cancer risk assessment. Therefore, a substantial research effort has been devoted to automate mammographic texture analysis, with the aim of ultimately incorporating such quantitative measures into breast cancer risk assessment models. In this paper, we review current and emerging approaches in this field, summarizing key methodological details and related studies using novel computerized approaches. We also discuss research challenges for advancing the role of parenchymal texture analysis in breast cancer risk stratification and accelerating its clinical translation.

**Conclusions:**

The objective is to provide a comprehensive reference for researchers in the field of parenchymal pattern analysis in breast cancer risk assessment, while indicating key directions for future research.

## Background

The incidence and mortality rates of breast cancer remain extremely high despite advances in screening and treatment [1]. In the USA, it is estimated that, in 2016, there will be 246,660 new cases of invasive breast cancer and 40,450 breast cancer deaths [2]. Therefore, better strategies are urgently needed to identify women at high risk for developing breast cancer who could benefit the most from supplemental screening and preventive therapies [3, 4].

Unfortunately, to date, the broadly available risk assessment models cannot identify high-risk women reliably within the general population. Current models predict either the risk of carrying a high-risk genetic mutation such as BRCA1/2 (e.g., Claus model, BOADICEA, and BRCAPRO) or the risk of developing breast cancer over time with or without such a mutation (e.g., Gail model, BOADICEA, Rosner-Colditz model, and Tyrer-Cuzick model) [5]. These models have only modest discriminatory capacity and continuing efforts are needed to improve these models at the individual level [6]. In addition, genetic susceptibility models are only useful in the familial setting (where cancer pedigree history is known) and are not of relevance to the general population where the great majority of women have no relevant family history. Therefore, in striving to tailor breast cancer screening recommendations for *the individual woman* [7] it is crucial to develop more accurate risk assessment models that can be easily adopted in routine clinical practice.

While mammography remains the cornerstone of early breast cancer detection [8], it also provides a readily accessible method to assess the distribution of fatty and dense, or fibroglandular (stromal and epithelial), tissues in the breast. In x-ray imaging, fatty tissue appears radiographically lucent, or darker, and dense tissue is radio-opaque, or brighter. Mammographic percent density (PD), a measure of the relative amount of fibroglandular tissue within the breast, has been shown to be related to screening sensitivity and specificity and has also been established as a strong independent risk factor for breast cancer [9–12]. Studies have repeatedly shown significant associations with breast cancer risk for both qualitative and quantitative breast density measures and a potential to improve cancer risk assessment models [13, 14]. Recent legislation in several US states mandates notification of breast density [15], and substantial research continues to be devoted to accurate measurement of this key biomarker and to its incorporation into risk prediction models [9, 16].

Compared to the global image measure of breast density, parenchymal texture descriptors can provide more refined, localized descriptors to characterize the complexity as well as the morphological distribution of the breast parenchymal patterns. Breast density measures are generally dichotomous, or each area or voxel of breast density measured in the mammogram is compared to a threshold of “dense” or “not dense” without reflecting the broader range and spatial distribution of the various breast parenchymal elements. Parenchymal *textural* features have been proposed as not only imaging markers that could identify parenchymal changes associated with breast cancer development [17–19], but also with subtypes and grading of subsequent breast malignancies [20–22]. In addition, there is growing evidence in support of textural features of the breast parenchyma reflecting inherent, independent, biologic risk factors associated with cancer development, and this may thus have the potential to augment breast density in assessing an individual woman’s risk of developing cancer [23–25]. Therefore, efforts to incorporate breast parenchymal texture analyses in breast cancer risk assessment have recently also gained substantial momentum.

This article reviews approaches to quantitate mammographic textural features and methods to incorporate these features into breast cancer risk assessment models, focusing primarily on novel computerized approaches. A systematic review of the literature in PubMed was performed to identify all original articles published up to April 2016 that evaluated computational measures of mammographic texture in breast cancer risk assessment. The following keywords were used in combination: “texture” or “parenchymal patterns” or “image features”, “mammography” or “mammogram”, and “breast cancer risk” or “mammographic risk”. To broaden the search, the “related articles” function provided in PubMed was also used, and all articles and citations obtained were reviewed. The references from all the articles identified were also examined for further relevant studies. The last search was conducted on 29 April 2016. Studies not considered relevant to the scope of the review were excluded; other exclusion criteria included: study not published in the English language, full text not available, letter to the editor, and duplicate publication. In the rest of this manuscript, we summarize key methodological details and evaluation results from the 44 research papers identified by the search and discuss future challenges in this promising research field.

## Mammographic texture analysis using automatically extracted features

The value of characterizing the mammographic texture of the breast parenchyma in breast cancer risk estimation was originally demonstrated in the pioneering studies of Wolfe [26, 27], Boyd et al. [28–30], Gram et al. [31], and Brisson et al. [32], proposing visually assessed, qualitative or quantitative classifications which were based on the extent and the characteristics of breast densities in a mammogram. These early approaches have been used by several groups, generally reporting elevated risks among women with more complex parenchymal tissue patterns [33–45]. Nevertheless, these studies also observed increased heterogeneity and low reproducibility in corresponding risk estimates due to subjectivity and inter-observer variation in visual appraisal of the mammogram [33–45]. By introducing computerized texture features to automate the characterization of breast parenchymal patterns, later studies addressed the limitations of visual classifications and re-established the potential of texture descriptors in breast cancer risk assessment [46–50]. Since then, this research field has continuously been evolving. A variety of quantitative methodologies have been developed, involving different techniques to sample the breast and multiple texture descriptors to characterize the texture properties of the sampled regions of interest (ROIs) from cranio-caudal (CC) and mediolateral-oblique (MLO) view mammograms (Table 1).

**Table 1:** Key studies in automated parenchymal texture analysis for breast cancer risk assessment

Study		Mammograms		Dataset		Breast sampling		Texture features				
Year	Participating institutions	F/D	View	A	B	S1	S2	T1	T2	T3	T4	T5
Distinguishing or predicting cancer cases from controls												
Byng et al. (1997) [60]	University of Toronto, Sunnybrook Health Science Centre, Ontario Cancer Institute	F	CC	354^P^	354	x		x			x	
Torres-Mejia et al. (2005) [72]	LSHTM, Guy’s Hospital, UNAM, IPOFG	F	CC/MLO	111^P^	3100	x		x			x	
Wu et al. (2008) [76]	University of Michigan	F	CC	128^C^	549	x				x		
Manduca et al. (2009) [66]	Mayo Clinic, Moffitt	F	CC/MLO	246^P^	522	x		x	x	x	x	x
Wei et al. (2011) [73]	University of Michigan	F	CC	136^C^	246	x				x		
Nielsen et al. (2011) [61]	University of Copenhagen, Nordic Bioscience, Delft University of Technology, RadboudUMC, Mayo Clinic	F	MLO	245^P^	250	x					x	x
Brandt et al. (2011) [74]	University of Copenhagen, RadboudUMC, Synarc Imaging Technologies	F	MLO	245^P^	245		x				x	
Häberle et al. (2012) [56]	Erlangen University Hospital, Fraunhofer Institute for Integrated Circuits IIS, IMPRS, UCLA	F	CC	864^C^	418	x		x	x	x	x	x
Li et al. (2012) [84]	University of Chicago	D	CC	75^C^	328	x		x	x		x	x
Chen et al. (2014) [75]	University of Manchester	D	MLO	50^C^	50		x					x
Nielsen et al. (2014) [64]	​University of Copenhagen, Nordic Bioscience, Biomediq, RadboudUMC, Mayo Clinic	F	CC/MLO	471^P,C^	692	x					x	x
Li et al. (2014) [71]	University of Chicago	D	CC	75^C^	328	x		x	x			x
Karemore et al. (2014) [89]	​University of Copenhagen, RadboudUMC	F	MLO	245^P^	250		x				x	x
Zheng et al. (2015) [51]	University of Pennsylvania	D	MLO	106^C^	318		x	x	x	x	x	
Sun et al. (2015) [53]	University of Texas, China Northeastern University, University of Oklahoma, TTUHS, Guiyang Medical University	D	CC	141^P^	199		x	x	x			x
Tan et al. (2015) [77]	University of Texas, University of Oklahoma, University of Pittsburgh	D	CC/MLO	812^P^	1084	x		x	x	x	x	
Tan et al. (2015) [78]	University of Oklahoma, University of Pittsburgh	D	CC/MLO	430^P^	440	x			x	x	x	x
Predicting the risk of carrying a high-risk genetic mutation												
Huo et al. (2000) [80]	University of Chicago	F	CC	15	143	x		x	x			x
Huo et al. (2002) [55]	University of Chicago, University of Pennsylvania	F	CC	30	142	x		x	x			x
Li et al. (2004) [54]	University of Chicago, University of Pennsylvania	F	CC	30	60	x		x	x		x	x
Li et al. (2005) [81]	University of Chicago	F	CC	30	142	x		x	x		x	x
Li et al. (2007) [82]	University of Chicago	F	CC	30	142	x					x	
Li et al. (2008) [83]	University of Chicago	F	CC	30	142	x						x
Li et al. (2012) [84]	University of Chicago	D	CC	53	328	x		x	x		x	x
Li et al. (2014) [71]	University of Chicago	D	CC	53	328	x		x	x			x
Gierach et al. (2014) [85]	University of Chicago, NCI-NIH, Washington Radiology Associates, Genentech, USUHS, UCL, WRNMC, Westat Inc.	F	CC	137	100	x		x	x		x	x

The Table describes the image data used in each study, including type of mammograms and dataset size, as well as methodological details for the computerized texture analysis, the technique of breast sampling, and algorithm implementation of texture features

*IMPRS* International Max Planck Research School for Optics and Imaging, *IPOFG* Instituto Português de Oncologia Francisco Gentil, *LSHTM* London School of Hygiene and Tropical Medicine, *Moffitt* Moffitt Cancer Center and Research Institute, *NCI-NIH* National Cancer Institute, National Institutes of Health, *RadboudUMC* Radboud University Nijmegen Medical Centre, *TTUHS* Texas Tech University Health Sciences, *UCL* University College London, *UCLA* University of California at Los Angeles, *UNAM* Universidad Nacional Autónoma de México, *USUHS* Uniformed Services University of the Health Sciences, *WRNMC* Walter Reed National Military Medical Center

Mammograms: *F* Digitized screen-film, *D* Full-field digital, *CC* cranio-caudal, *MLO* mediolateral-oblique; Dataset: *A* cancer cases (^P^prior, unaffected, images, ^C^images from the contralateral, unaffected, breast at the time of cancer diagnosis) or other high-risk population (i.e., BRCA1/2 carriers), *B* controls; Breast sampling: *S1* retro-areolar region or the entire breast/dense tissue as a single region of interest (ROI), *S2* multiple ROIs covering the entire breast; Types of texture features: *T1* gray-level histogram, *T2* co-occurrence, *T3* run-length, *T4* structural/pattern, *T5* multi-resolution/spectral

**Fig. 1:**
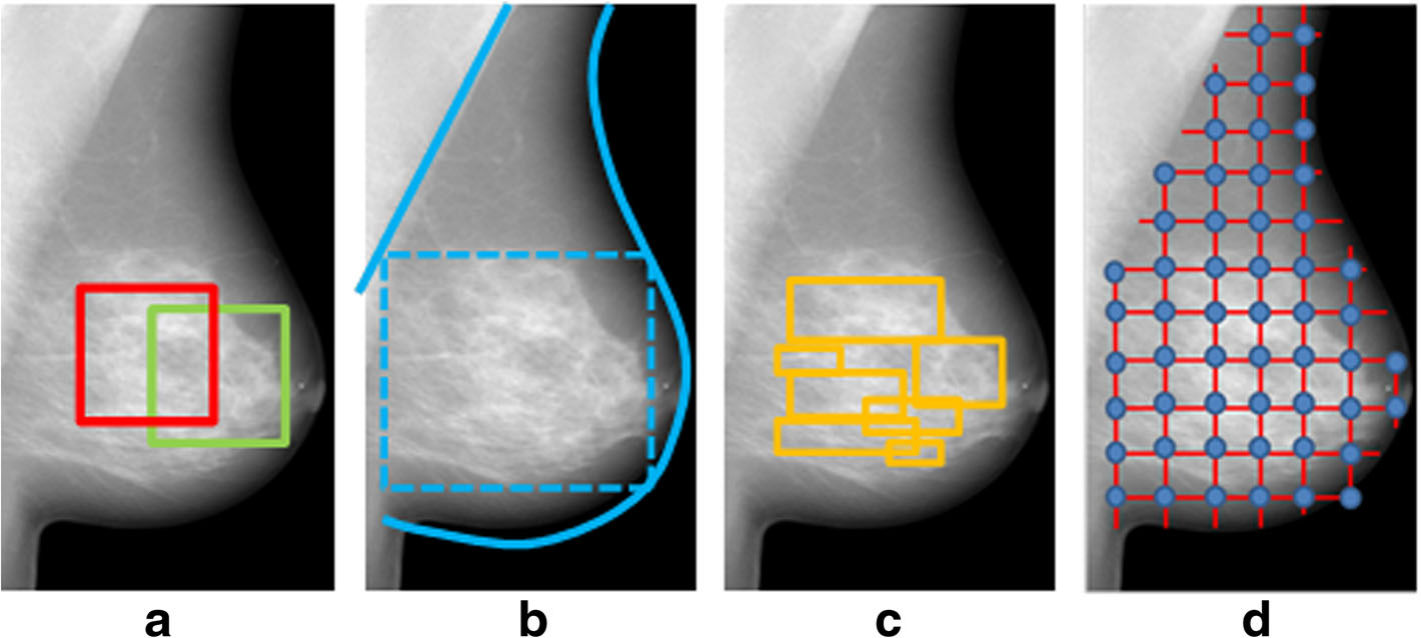
Regions of interest (ROIs) used in texture analysis. **a** single ROIs selected in the retro-areolar breast area, **b** the entire breast and the largest rectangular box inscribed within the breast, studied as single ROIs, **c** multiple ROIs at multiple scales of density, and **d** multiple ROIs defined by a lattice covering the entire breast

In most studies, texture analysis has been performed within a single ROI in the breast (Table 1). This single ROI is usually placed in the retroareolar breast area, while, in some cases, it can be a larger region corresponding to the entire breast or to the largest rectangular box inscribed within the breast (Fig. 1a and b). In an attempt to capture the granularity and heterogeneity of the parenchymal texture within the breast, more recent studies have estimated texture in multiple ROIs throughout the breast (Fig. 1c and d). A lattice-based strategy which splits the entire breast into multiple square patches was proposed by Zheng et al. [51, 52] showing that, with respect to single ROI methodologies, this breast sampling technique may improve risk assessment, with performance being maximized when smaller patches (6.3 × 6.3 mm^2^) are used. Multiple ROIs defined at various scales of breast tissue density were used by Sun et al. [53], where it was shown that fusing features from different density scales may prove to be another effective way to enhance the cancer prediction performance.

**Fig. 2:**
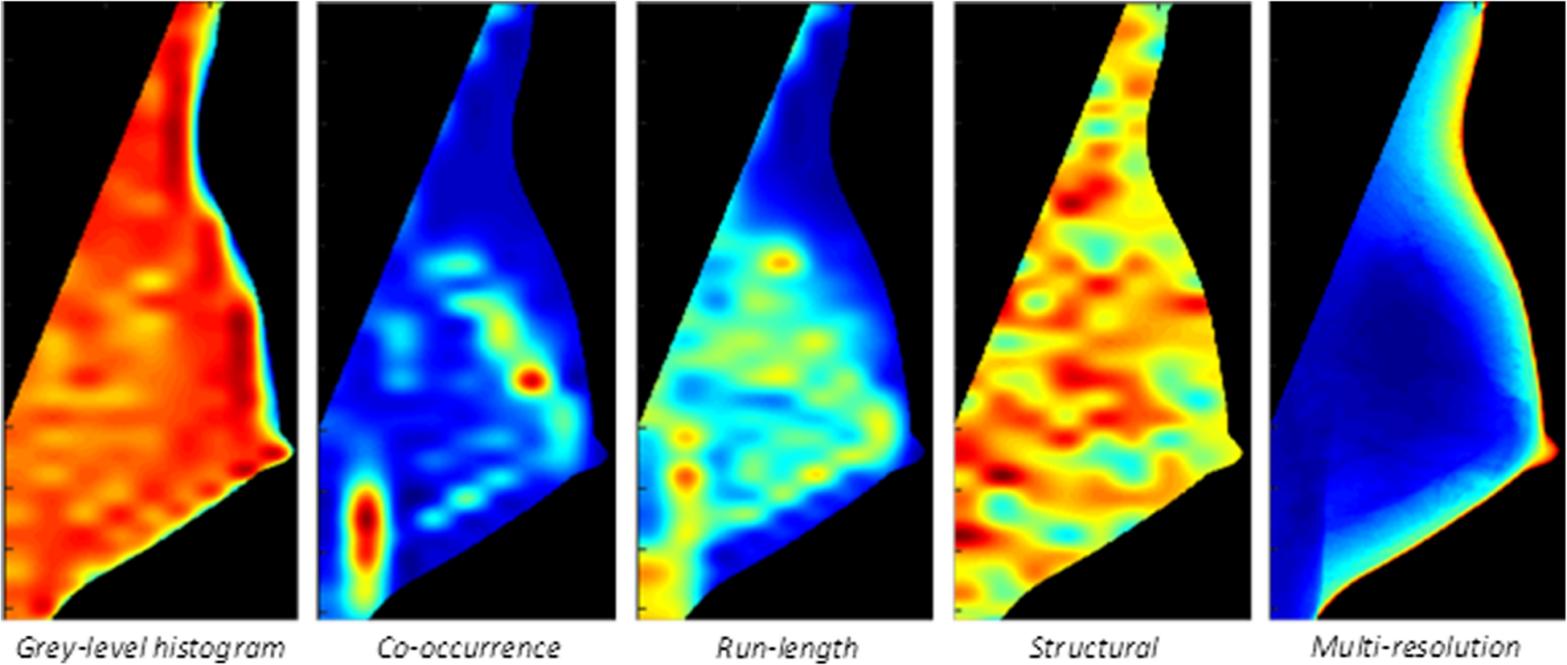
Characterization of parenchymal patterns using computerized texture analysis. Examples of feature maps showing the distribution of texture values in the breast, generated by the application of the lattice-based strategy of Zheng et al. [51] to an MLO-view full-field digital mammogram. (**a**) Grey-level histogram, (**b**) Co-occurrence, (**c**) Run-length, (**d**) Structural, and (**e**) Multi-resolution

The texture descriptors used in breast cancer risk assessment to date can be broadly classified into five feature groups (Table 2), each of which reveals different aspects of the mammographic texture (Fig. 2): 1) grey-level intensity/histogram features [54–56]; 2) co-occurrence (Haralick/Markovian) descriptors [57]; 3) run-length features [58, 59]; 4) structural/pattern measures [46, 60–65]; and 5) multi-resolution/spectral features [53, 61, 64, 66, 67]. Gray-level intensity histogram features are common first-order statistics which describe the distribution of gray-level intensity within the breast tissue. The co-occurrence features also consider the spatial relationships of pixel intensities in different directions and are based on the gray-level co-occurrence matrix (GLCM) which encodes the relative frequency of neighboring intensity values. Run-length features capture the coarseness of texture in specified directions by measuring strings of consecutive pixels (i.e., runs) which have the same gray-level intensity along specific linear orientations. Fine textures tend to contain more short runs with similar gray-level intensities, while coarse textures have longer runs with different gray-level intensities. Structural features capture the architectural composition of the parenchyma by characterizing the tissue complexity, the directionality of flow-like structures in the breast, and intensity variations between central and neighboring pixels. Finally, multi-resolution/spectral features use spatial frequency transforms, such as Fourier, wavelet/Gabor, and the Power spectrum, to characterize intrinsic periodic texture structures that repeat over multiple scales.

**Table 2:** Parenchymal texture descriptors for breast cancer risk assessment; texture descriptors which have been examined in association with breast cancer risk, classified to five feature groups

Grey-level histogram features [51, 53–56, 60, 66, 71, 72, 77, 80, 81, 84, 85]			
min intensity	skewness	5^th^ percentile	energy
max intensity	kurtosis	5^th^ percentile mean	root mean square variation
standard deviation	entropy	95^th^ percentile	
mean intensity	sum intensity	95^th^ percentile mean	
Co-occurrence features [51, 53–56, 66, 71, 77, 78, 80, 81, 84, 85]			
cluster shade	entropy	inverse difference moment	difference entropy
correlation	inertia	sum variance	homogeneity
Haralick correlation	difference moment	sum average	product moment
energy	coarseness	difference variance	triangular symmetry
Run-length measures [51, 56, 66, 73, 76–78]			
long run emphasis	gray-level non-uniformity	high gray level run emphasis	run percentage
short run emphasis	run-length non-uniformity	low gray level run emphasis	number of runs
Structural/Pattern measures [51, 54, 56, 60, 61, 64, 66, 72, 74, 77, 78, 81, 82, 84, 85, 89]			
fractal dimension	local binary pattern	Hessian matrix	Weber local descriptors
lacunarity	Law’s masks	edge enhancing index	directional gradient
Multi-resolution/Spectral features [53–56, 61, 64, 66, 71, 75, 78, 80, 81, 83–85, 89]			
Fourier power spectrum	wavelet/Gabor	Gaussian Kernels	power-law spectrum

## Towards a new breast cancer risk assessment paradigm based on mammographic texture descriptors

The proposed methodologies have been applied primarily to digitized film-screen mammograms and more recently on full-field digital mammograms. Texture descriptors have been evaluated in a few prospective and a larger number of retrospective case–control studies, where their discriminatory capacity in breast cancer prediction was typically assessed in terms of the area under the ROC curve (AUC) measuring their ability to distinguish between cancer cases and controls (Table 3). The potential of mammographic texture in breast cancer risk assessment has also been investigated in studies with BRCA1/2 mutation carriers, where the AUC was evaluated in terms of the performance of the texture features in predicting a woman’s risk of carrying this high-risk genetic mutation. Although hereditary breast cancers account for 5–10 % of incident breast cancers, women who inherit a mutated form of the BRCA1/2 gene have up to 87 % risk of developing breast cancer by the age of 70 years [68]. As such, and considering that mammographic PD has not been associated with BRCA1/2 mutation status [69–71], the ability of texture to identify potential BRCA1/2 carriers could have important value in risk stratification.

**Table 3:** Breast cancer prediction capacity of automated characterization of the parenchymal patterns

Study		Dataset			Model	Discriminatory capacity (AUC)					
Year	Participating Institutions	A	B	m		^$^Texture	^$^PD	^$^Texture + PD	^Texture	^PD	^Texture + PD
Distinguishing between cancer cases and healthy women											
Wu et al. (2008) [76]	University of Michigan	128^C^	549	No	LDA^CV^	0.73					
Manduca et al. (2009) [66]	Mayo Clinic, Moffitt	246^P^	522	Yes	LR^CV^	[0.58, 0.60]	0.58		Age, BMI		
									[0.61, 0.62]	0.60	[0.62, 0.63]
Wei et al. (2011) [73]	University of Michigan	136^C^	246	No	LDA	0.74*	0.61	0.76	Age, BMI, family history of breast cancer, #of previous breast biopsies		
											0.78
Nielsen et al. (2011) [61]	University of Copenhagen, Nordic Bioscience, Delft University of Technology, RadboudUMC, Mayo Clinic	245^P^	250	No	LR^CV^	0.63	0.60	0.66*			
Brandt et al. (2011) [74]	University of Copenhagen, RadboudUMC, Synarc Imaging Technologies	245^P^	245	Yes	kNN^CV^	0.63	0.56				
Häberle et al. (2012) [56]	Erlangen University Hospital, Fraunhofer Institute for Integrated Circuits IIS, IMPRS, UCLA	864^C^	418	Yes	LR^CV^	0.75	0.51	0.75	Age, BMI, family history of breast cancer, parity, age at first term pregnancy		
									0.79	0.66	0.79
Li et al. (2012) [84]	University of Chicago	75^C^	328	No	BANN^CV^	0.73					
Li et al. (2012) [84]	University of Chicago	67^C^	268	Yes	BANN^CV^	0.70					
Chen et al. (2014) [75]	University of Manchester	50^C^	50	No	LR	0.71	0.62	0.68			
Nielsen et al. (2014) [64]	UCPH, Nordic Bioscience, Biomediq, RadboudUMC, Mayo Clinic	245^P^	250	No	LR^CV^				Age, BMI, menopause, hormonal use		
									0.60	0.63	0.66
Nielsen et al. (2014) [64]	UCPH, Nordic Bioscience, Biomediq, RadboudUMC, Mayo Clinic	226^C^	442	Yes	LR^CV^				Age, BMI, menopause, hormonal use		
									0.61	0.63	
Li et al. (2014) [71]	University of Chicago	67^C^	268	Yes	BANN^CV^	0.70*	0.57	0.68			
Karemore et al. (2014) [90]	UCPH, RadboudUMC	245^P^	250	Yes	kNN^CV^	0.59					
Zheng et al. (2015) [51]	University of Pennsylvania	106^C^	318	Yes	LR^CV^	0.85*	0.59	0.86			
Sun et al. (2015) [53]	University of Texas, China Northeastern University, University of Oklahoma, TTUHS, Guiyang Medical University	141^P^	199	No	SVM^CV^	0.73			Age, BMI, family history of breast cancer, hormonal use, age at first term pregnancy		
									0.77		
Tan et al. (2015) [77]	University of Texas, University of Oklahoma, University of Pittsburgh				ANN^CV^				age		
		812^P^	1084	No		0.71			0.78		
Tan et al. (2015) [78]	University of Oklahoma, University of Pittsburgh	430^P^	440	No	ANN^CV^	[0.64, 0.73]					
Predicting the risk of carrying a high-risk genetic mutation											
Huo et al. (2000) [80]	University of Chicago	15	143	No	LDA	[0.59, 0.82]					
Huo et al. (2000) [80]	University of Chicago	15	30	Yes	LDA	[0.53, 0.87]					
Huo et al. (2002) [55]	University of Chicago, University of Pennsylvania	30	142	No	LDA	0.91					
Huo et al. (2002) [55]	University of Chicago, University of Pennsylvania	30	60	Yes	LDA	0.92					
Li et al. (2004) [54]	University of Chicago, University of Pennsylvania	30	60	Yes	LDA^CV^	[0.69, 0.92]					
Li et al. (2005) [81]	University of Chicago	30	142	No	ROCA	[0.66, 0.86]					
Li et al. (2005) [81]	University of Chicago	30	60	Yes	ROCA	[0.67, 0.86]					
Li et al. (2007) [82]	University of Chicago	30	142	No	ROCA^CV^	[0.74, 0.93]					
Li et al. (2007) [82]	University of Chicago	30	60	Yes	ROCA^CV^	[0.77, 0.91]					
Li et al. (2008) [83]	University of Chicago	30	142	No	ROCA	0.90					
Li et al. (2008) [83]	University of Chicago	30	60	Yes	ROCA	0.89					
Li et al. (2012) [84]	University of Chicago	53	328	No	BANN^CV^	0.82					
Li et al. (2012) [84]	University of Chicago	34	136	Yes	BANN^CV^	0.81					
Li et al. (2014) [71]	University of Chicago	34	136	Yes	BANN^CV^	0.81*	0.53	0.81			
Gierach et al. (2014) [85]	University of Chicago	137	100	No	BANN^CV^	0.68	0.59	0.72			
Gierach et al. (2014) [85]	University of Chicago, NCI-NIH, Washington Radiology Associates, Genentech, USUHS, UCL, WRNMC, Westat Inc.	126	89	Yes	BANN^CV^	0.71	0.55	0.72			

Area under the ROC curve (AUC) achieved by risk assessment models when fed with mammographic texture and/or density measures

*IMPRS* International Max Planck Research School for Optics and Imaging, *Moffitt* Moffitt Cancer Center and Research Institute, *NCI-NIH* National Cancer Institute, National Institutes of Health, *RadboudUMC* Radboud University Nijmegen Medical Centre, *TTUHS* Texas Tech University Health Sciences, *UCL* University College London, *UCLA* University of California at Los Angeles, *USUHS* Uniformed Services University of the Health Sciences, *WRNMC* Walter Reed National Military Medical Center

Dataset: *A* cancer cases (^P^prior unaffected images, ^C^images from the contralateral, unaffected, breast at the time of cancer diagnosis) or other high-risk population (i.e., BRCA1/2 carriers), *B* controls, *m* matched subgroups; Model: *LDA* Linear Discriminant Analysis, *LR* Logistic Regression, *kNN* k-nearest neighbors, *BANN* Bayesian Artificial Neural Network, *ANN* Artificial Neural Network, *ROCA* Receiver Operating Characteristic Analysis. *PD* percent density. ^CV^cross-validated models; ^$^unadjusted models; ^models adjusted for established risk factors; *statistically significant from ^$^PD at < 0.05

### Associations of parenchymal texture with breast cancer in case–control studies

Byng et al. [60] were the first to evaluate automatically calculated parenchymal texture descriptors directly as independent risk factors for breast cancer. The authors reported on data from a prospective case–control study using 354 incident cases diagnosed with histologically verified invasive breast carcinoma at least 1 year after their entry in the Canadian National Breast Screening Study, and 354 age-matched controls with at least 7 years of negative follow-up. Two grey-level intensity histogram texture features were estimated in screen-film mammograms; specifically, skewness averaged over individual 6.2 × 6.2 mm^2^ patches in the breast and the fractal dimension estimated by considering the entire breast as a single ROI. For both features, the results showed moderate relative risk (RR) after adjustments for the effects of other risk factors, i.e., age at menarche, menopausal status, age at first time pregnancy, number of live births, family history of breast carcinoma, height, and weight (RR = 3.35 and RR = 3.35 for skewness and fractal dimension, respectively), while no additional contribution to risk was found in models that incorporated breast density measures. Similar conclusions were reported by Torres-Mejia et al. [72] who estimated the same texture features and lacunarity, a measure of the degree of structural variation in image intensities within the breast, from prospectively collected data of 111 breast cancer cases and 3100 controls.

The promising results of these early studies were followed by retrospective studies using more complex parenchymal texture descriptors [61, 64, 73–76]. Wei et al. [73] investigated the associations of breast cancer risk with run-length features, using two different implementations of run-length statistics: namely, the conventional approach for calculating the runs of pixels in one direction and an extension for the two-dimensional space [76]. The authors found that the run-length measures calculated in the retroareolar region of the breast could serve as an additional risk factor that could not be explained by established breast cancer risk factors (i.e., age, BMI, family history of breast cancer, and number of previous biopsies) and breast density. A mammographic texture resemblance (MTR) marker based on multi-scale Gaussian features was proposed by Nielsen et al. [61]. This marker demonstrated high case–control discriminatory performance (AUC = 0.60–0.63) in two independent cohorts within the Dutch screening program [61] and the Mayo Mammography Health Study [61, 64], while performance was optimized by an aggregate marker combining MTR with density measures (AUC = 0.66). Gaussian derivative features at multiple scales were examined in a cross-sectional study with MLO-view film mammograms of 245 cancer cases and 245 controls from the Nijmegen risk-assessment study [74]. In this work, derivative features were extracted using an anatomically oriented breast coordinate system and, compared to breast PD, demonstrated enhanced breast cancer prediction ability (AUC = 0.63 versus 0.56). Finally, a preliminary study on the dual-tree complex wavelet transform showed that wavelet features alone may have value in risk assessment [75].

In an attempt to identify highly discriminative texture descriptors from multiple feature groups and develop optimal combinations that maximize the case–control classification performance, research groups have also explored comprehensive sets of multi-parametric features reflecting various aspects of mammographic texture [51, 53, 56, 66, 77, 78]. Following an evaluation of more than 1000 co-occurrence, run-length, Laws, wavelet, and Fourier features in prior film mammograms of 246 cases and 522 controls, Manduca et al. [66] identified individual features which, when estimated at a coarse scale of a single ROI covering the entire breast, provided strong prediction for future breast cancer (odds ratio per 1 SD = 1.36–1.50, AUC = 0.61–0.62). In another retrospective study with 864 cancer cases and 418 controls, a three-step variable selection process separated 46 highly discriminative features from a total number of 470 features initially calculated [56]. When fed to multivariable logistic regression models adjusted for established breast cancer risk factors, these features demonstrated an AUC of 0.79 and an odds ratio of 2.88, while the additional inclusion of breast PD did not lead to any further performance improvement.

Promising results from rich feature sets were also recently reported for digital mammograms. In a study with CC-view digital mammograms of 141 cases and 199 controls, a total number of 765 features were computed from ROIs defined at multiple density scales [53]. From these features, an optimal set of 12 features was selected and yielded an AUC of 0.73 in separating the two study subgroups using a support vector machine classifier. Zheng et al. [51] retrospectively analyzed MLO-view digital mammograms of 106 cases and 318 controls, where 30 candidate features were extracted from multiple adjacent ROIs covering the entire parenchyma. The authors showed a collective discriminatory capacity of AUC = 0.85, with the fractal dimension, run-length, co-occurrence, and gray-level histogram features being more frequently selected than local binary and edge-enhancing index features in classification models. Furthermore, preliminary comparisons of the parenchymal patterns of estrogen-receptor positive (ER+) and negative (ER–) cancer cases measured with the same methodology [51] showed that subtype-specific breast cancer risk assessment based on mammographic textures may also be feasible [79]. Finally, to assess the combined discriminatory ability of texture analysis in CC and MLO views, Tan et al. [78] designed an artificial neural network model to fuse the features extracted from the two views. Following an evaluation of 79 features calculated from a single ROI, corresponding to either the entire breast or the dense tissue areas of the breast, on 430 cases and 440 controls, the highest performance of the proposed fusion model (AUC = 0.73) was obtained for the run-length features of the dense tissue. The authors also demonstrated a classification performance of similar magnitude (AUC = 0.71) for the same fusion model when applied on texture features of the entire breast for a larger dataset of 821 cancer cases and 1084 controls [77].

### Assessing the risk of carrying a high-risk gene mutation

The potential of mammographic texture in breast cancer risk assessment has also been demonstrated in studies with BRCA1/2 carriers, where texture features from a single 25.6 × 25.6 mm^2^ retro-areolar ROI in CC mammographic views were shown to predict a woman’s risk of carrying this high-risk genetic mutation. The first study addressing this topic extracted a comprehensive feature set of grey-level intensity statistics, co-occurrence features, and multi-scale texture measures based on Fourier transform analysis [80]. In film mammograms of 30 BRCA1/2 carriers and 142 low-risk women, most features demonstrated high individual discriminatory capacity (AUC > 0.68), while the collective performance of the features that were deemed significant in multivariable models raised AUC values of 0.91 and 0.92 in the entire database and in an age-matched subgroup, respectively [55]. Using the same image dataset, the authors also showed a promising individual classification performance for structural measures such as edge frequency (AUC = 0.78) [81], for different implementations of the fractal dimension (AUC = 0.74–0.93) [81, 82], and for power law spectral analysis (AUC = 0.90) [83].

These results were recently replicated and validated in datasets with digital mammograms [71, 84] and larger numbers of high-risk women [71, 84, 85]. A similar design of texture analysis in the retroareolar breast region combined with a Bayesian Artificial Neural Network (BANN) for the classification task was applied to 1) film mammograms of 137 mutation carriers and 100 low-risk women [85], and 2) digital mammograms of 53 mutation carriers, 75 women with unilateral cancer, and 328 low-risk women [71, 84]. The first analysis conferred a two-fold increase in the odds of predicting BRCA1/2 mutation status, and an AUC of 0.68 for texture features alone and 0.72 for the features plus breast PD [85]. In the second analysis, AUC values of 0.82 and 0.73 were obtained between mutation carriers and low-risk women, and between unilateral cancer and low-risk women, respectively [84]; these evaluation results were also retained in age-matched subgroup analysis (0.81 and 0.70, respectively) [84] without any significant improvement from the inclusion of breast PD (0.81 and 0.68, respectively) [71].

### Beyond established risk factors in breast cancer risk assessment

A comparison of the evaluation results published to date (Table 3), focusing primarily on cross-validated experiments, suggests that more comprehensive sets of multi-parametric texture features [51, 53, 54, 56, 71, 77, 84] may be more effective in predicting breast cancer than a single feature group. However, the literature lacks extensive comparative studies on the same datasets and generalized conclusions should, therefore, be limited. While the implementation of texture analysis, including both the location and size of ROIs [51, 52, 54] and the specific texture measures, appears to have an effect on texture classification performance, all studies have consistently shown the highly promising, independent role of automated texture analysis in breast cancer risk assessment. Specifically, parenchymal texture descriptors have demonstrated a strong cross-validated ability in predicting both risk for breast cancer (0.58 ≤ AUC ≤ 0.85) and BRCA1/2 mutation status (0.53 ≤ AUC ≤ 0.93). Moreover, texture performance has been shown to be either comparable or significantly higher than the performance of breast PD (0.51 ≤ AUC ≤ 0.62 and 0.53 ≤ AUC ≤ 0.59, respectively), as reported in studies where texture and density measures were comparatively evaluated on the same datasets [51, 56, 61, 66, 71, 73–75, 85].

In addition, a number of related findings suggest that texture analysis is able to provide complementary information about a woman’s risk of developing breast cancer which cannot be captured by breast PD and other established risk factors. Texture descriptors have been weakly or moderately correlated with breast PD [55, 61, 64, 71–73, 85–87], and weakly correlated with risk factors as reflected in the Gail and Claus risk scores [80, 86]. In addition, texture descriptors deemed as strong predictors of breast cancer retained significance when breast PD, age, BMI, family history of breast cancer, parity, age at first term pregnancy, number of previous breast biopsies, menopause, and hormonal use, all shown to be associated with breast cancer risk, were simultaneously considered in classification models (Table 3). Finally, with age-matched datasets or model adjustments for age, most studies evaluating the capacity of parenchymal texture features in risk assessment have ruled out possible confounding due to differences in age, a major breast cancer risk factor, thereby showing a strong potential for computerized texture descriptors in augmenting breast cancer risk assessment.

## Future directions

Moving forward, experiments evaluating the relative performance of different implementations of texture analysis, using the same evaluation methodology (i.e., dataset and classification model), are necessary to develop more robust and reproducible quantitative mammographic phenotypes of breast cancer risk. Future studies to test the incremental value added by computerized textural measures in predicting breast cancer will require: (a) the design of large age-matched datasets; (b) the selection of an effective classification model, where different previously used models (Table 3) could be comparatively examined; (c) model adjustments to rule out possible confounding due to differences in major risk factors; and (d) validation of the classification performance in independent datasets.

In an attempt to add an anatomical meaning in texture analysis which may also give additional discrimination power to feature classification, increasing attention is currently given to the incorporation of breast anatomy in texture analysis pipelines. Brandt et al. [74] first introduced an anatomically oriented breast coordinate system which allows for anatomical correspondences across mammograms of the same woman or different women. In preliminary analyses using the proposed coordinate system, the authors have demonstrated that anatomy-driven Gaussian derivative features are able to (a) effectively separate cancer cases and controls [74], (b) quantify the effect of hormone replacement therapy as a change in the breast parenchymal patterns [88], and (c) demonstrate specific regions of the breast parenchyma where breast cancer risk is mainly expressed [89]. More recently, Gastounioti et al. [90–92] showed that the discriminatory capacity of texture descriptors is further enhanced by an anatomy-driven polar grid for anatomical breast sampling and a breast-anatomy-weighted texture signature which considers the spatial position and the underlying tissue composition of individual ROIs to summarize the parenchymal texture properties of the breast.

Another emerging technology is deep learning [93], which may prove a valuable addition in texture analysis for breast cancer risk assessment [94–96]. Deep learning involves automated learning, from raw image data, of hierarchical representations useful for pattern detection and classification, in a supervised mode via neural networks with multiple hidden layers or in an unsupervised mode via autoencoders. The few available studies which have applied deep learning in the particular field show a promising role in risk scoring (AUC = 0.61–0.65) [94, 96]. Further, preliminary comparisons against two previously presented methodologies with handcrafted texture features [56, 64] suggest that it may be better to “let the data speak” instead of modeling prior assumptions [94]. Additional experimentation with deep learning, as well as future comparisons with the state-of-the-art texture analysis techniques, is warranted to better explore the potential of this novel technology.

Digital breast tomosynthesis (DBT), an emerging x-ray technology [97] in which quasi three-dimensional (3D) images are reconstructed from a limited number of low-dose x-ray source projections [98, 99], is increasingly being implemented clinically due to improvements in sensitivity and specificity compared to imaging with digital mammography alone [100]. By imaging the breast in 3D, DBT alleviates the effect of tissue superimposition, offering superior tissue visualization, which in turn may allow for better characterization of the breast parenchyma compared to two-dimensional mammography [86, 87]. The extension of the parenchymal texture analysis descriptors for volumetric texture analysis in DBT is, therefore, an important future challenge towards developing superior texture features which can optimize image-driven breast cancer risk assessment.

Another challenging future step which would establish the predictive value of texture analysis is the validation of parenchymal texture measures in prospectively collected data. Large-scale studies involving multiple screening centers, imaging machines, and image acquisition settings are also of major importance towards validating their predictive capacity and robustness to heterogeneous image data [101]. Furthermore, the literature lacks large-scale longitudinal studies monitoring longitudinal changes in automated parenchymal texture descriptors over successive mammograms, which could elucidate the mechanisms of breast cancer development [11] and the causal relations between the texture risk scoring and breast cancer [102, 103]. Finally, crucial questions to be addressed in such rich datasets are the causes of inter-woman variation in mammographic parenchymal patterns [104, 105] and in the relation of texture risk markers to the subsequent location and grading of tumors, disease mortality, and treatment effects [20, 21].

The valuable risk markers provided by parenchymal texture analysis could also leverage the relatively new, yet promising, paradigms of radiomics [106] and radiogenomics [107] for breast cancer, aiming to convert breast images into comprehensive measurable data and to delve into the interaction between these data and genetic variants. These novel approaches may pave the way to revealing correlations with the genomic diversity present in breast cancer, understanding how biological processes are reflected in quantitative breast imaging phenotypes, and defining novel clinical biomarkers or biological surrogates [108–111], thus improving personalized breast cancer screening, monitoring, and treatment selection.

## Conclusions

Automated breast parenchymal texture analysis has the potential to elucidate imaging phenotypes of breast cancer risk, which is valuable in accelerating the translation of individualized risk stratification into routine breast cancer screening and prevention strategies. Future work addressing technical challenges in this field and large prospective studies are expected to further enhance and establish the predictive value of parenchymal texture measures for inclusion in breast cancer risk assessment models in clinical practice.

## Abbreviations

3D: Three-dimensional
AUC: Area under the ROC curve
CC: Cranio-caudal
DBT: Digital breast tomosynthesis
ER–: Estrogen receptor negative
ER+: Estrogen receptor positive
GLCM: Grey-level co-occurrence matrix
MLO: Medio-lateral oblique
MTR: Mammographic texture resemblance
PD: Percent density
ROI: Region of interest
RR: Relative risk

## References

[1] Ferlay J, Soerjomataram I, Ervik M, Dikshit R, Eser S, Mathers C (2013). GLOBOCAN 2012 v1.0, cancer incidence and mortality worldwide: IARC CancerBase No. 11 Lyon.

[2] Cancer facts and figures 2016 Atlanta, GA: American Cancer Society; 2016. http://www.cancer.org/research/cancerfactsstatistics/cancerfactsfigures2016/. Accessed 8 Mar 2016

[3] Hall P, Easton D (2013). Breast cancer screening: time to target women at risk. Br J Cancer.

[4] Howell A, Astley S, Warwick J, Stavrinos P, Sahin S, Ingham S (2012). Prevention of breast cancer in the context of a national breast screening programme. J Int Med.

[5] Amir E, Freedman OC, Seruga B, Evans DG (2010). Assessing women at high risk of breast cancer: a review of risk assessment models. J Natl Cancer Inst.

[6] Gail MH, Mai PL (2010). Comparing breast cancer risk assessment models. J Natl Cancer Inst.

[7] Onega T, Beaber EF, Sprague BL, Barlow WE, Haas JS, Tosteson AN (2014). Breast cancer screening in an era of personalized regimens: a conceptual model and National Cancer Institute initiative for risk-based and preference-based approaches at a population level. Cancer.

[8] McDonald ES, Clark AS, Tchou J, Zhang P, Freedman GM (2016). Clinical diagnosis and management of breast cancer. J Nucl Med.

[9] Chen J-H, Gulsen G, Su M-Y (2015). Imaging breast density: established and emerging modalities. Transl Oncol.

[10] Ng K-H, Lau S (2015). Vision 20/20: Mammographic breast density and its clinical applications. Med Phys.

[11] Sherratt MJ, McConnell JC, Streuli CH (2016). Raised mammographic density: causative mechanisms and biological consequences. Breast Cancer Res.

[12] McCormack VA, dos Santos Silva I (2006). Breast density and parenchymal patterns as markers of breast cancer risk: a meta-analysis. Cancer Epidemiol Biomarkers Prev.

[13] Brentnall AR, Harkness EF, Astley SM, Donnelly LS, Stavrinos P, Sampson S (2015). Mammographic density adds accuracy to both the Tyrer-Cuzick and Gail breast cancer risk models in a prospective UK screening cohort. Breast Cancer Res.

[14] Tice JA, Cummings SR, Smith-Bindman R, Ichikawa L, Barlow WE, Kerlikowske K (2008). Using clinical factors and mammographic breast density to estimate breast cancer risk: development and validation of a new predictive model. Ann Intern Med.

[15] Are You Dense Advocacy. D.E.N.S.E. State Efforts. http://areyoudenseadvocacy.org/. Accessed 1 June 2016.

[16] Abdolell M, Tsuruda K, Lightfoot CB, Payne JI, Caines J, Iles SE (2016). Utility of relative and absolute measures of mammographic density versus clinical risk factors in evaluating breast cancer risk at time of screening mammography. Br J Radiol.

[17] Tan M, Zheng B, Ramalingam P, Gur D (2013). Prediction of near-term breast cancer risk based on bilateral mammographic feature asymmetry. Acad Radiol.

[18] Tan M, Zheng B, Leader J, Gur D (2016). Association between changes in mammographic image features and risk for near-term breast cancer development. IEEE Trans Med Imaging.

[19] Wang X, Lederman D, Tan J, Wang XH, Zheng B (2010). Computerized detection of breast tissue asymmetry depicted on bilateral mammograms: a preliminary study of breast risk stratification. Acad Radiol.

[20] Holm J, Humphreys K, Li J, Ploner A, Cheddad A, Eriksson M (2015). Risk factors and tumor characteristics of interval cancers by mammographic density. J Clin Oncol.

[21] Bae MS, Moon H-G, Han W, Noh D-Y, Ryu HS, Park I-A (2015). Early stage triple-negative breast cancer: imaging and clinical-pathologic factors associated with recurrence. Radiology.

[22] Sala E, Solomon L, Warren R, McCann J, Duffy S, Luben R (2000). Size, node status and grade of breast tumours: association with mammographic parenchymal patterns. Eur Radiol.

[23] Oza AM, Boyd NF (1992). Mammographic parenchymal patterns: a marker of breast cancer risk. Epidemiol Rev.

[24] Daye D, Keller B, Conant EF, Chen J, Schnall MD, Maidment AD (2013). Mammographic parenchymal patterns as an imaging marker of endogenous hormonal exposure: a preliminary study in a high-risk population. Acad Radiol.

[25] Saftlas AF, Szklo M (1987). Mammographic parenchymal patterns and breast cancer risk. Epidemiol Rev.

[26] Wolfe JN (1976). Breast patterns as an index for developing breast cancer. Am J Roentgenol..

[27] Wolfe JN (1976). Risk for breast cancer development determined by mammographic parenchymal pattern. Cancer.

[28] Boyd N, O'Sullivan B, Campbell J, Fishell E, Simor I, Cooke G (1982). Mammographic signs as risk factors for breast cancer. Br J Cancer.

[29] Boyd N, Byng J, Jong R, Fishell E, Little L, Miller A (1995). Quantitative classification of mammographic densities and breast cancer risk: results from the Canadian National Breast Screening Study. J Natl Cancer Inst.

[30] Boyd N, Jensen H, Cooke G, Han HL (1992). Relationship between mammographic and histological risk factors for breast cancer. J Natl Cancer Inst.

[31] Gram IT, Funkhouser E, Tabár L (1997). The Tabar classification of mammographic parenchymal patterns. Eur J Radiol.

[32] Brisson J, Merletti F, Sadowsky NL, Twaddle JA, Morrison AS, Cole P (1982). Mammographic features of the breast and breast cancer risk. Am J Epidemiol.

[33] Egan RL, Mosteller RC (1977). Breast cancer mammography patterns. Cancer.

[34] Krook PM, Carlile T, Bush W, Hall MH (1978). Mammographic parenchymal patterns as a risk indicator for prevalent and incident cancer. Cancer.

[35] Threatt B, Norbeck JM, Ullman NS, Kummer R, Roselle P (1980). Association between mammographic parenchymal pattern classification and incidence of breast cancer. Cancer.

[36] Tabár L, Dean PB (1982). Mammographic parenchymal patterns: risk indicator for breast cancer?. JAMA.

[37] Wolfe JN, Saftlas AF, Salane M (1987). Mammographic parenchymal patterns and quantitative evaluation of mammographic densities: a case–control study. Am J Roentgenol.

[38] Saftlas AF, Wolfe JN, Hoover RN, Brinton LA, Schairer C, Salane M (1989). Mammographic parenchymal patterns as indicators of breast cancer risk. Am J Epidemiol.

[39] Myers L, McLelland R, Stricker C, Feig S, Martin J, Moskowitz M (1983). Reproducibility of mammographic classifications. Am J Roentgenol.

[40] Toniolo P, Bleich AR, Beinart C, Koenig KL (1992). Reproducibility of Wolfe's classification of mammographic parenchymal patterns. Prev Med.

[41] Goodwin PJ, Boyd NF (1988). Mammographic parenchymal pattern and breast cancer risk: a critical appraisal of the evidence. Am J Epidemiol.

[42] Witt I, Hansen HS, Brünner S (1984). The risk of developing breast cancer in relation to mammography findings. Eur J Radiol.

[43] Warner E, Lockwood G, Tritchler D, Boyd N (1991). The risk of breast cancer associated with mammographic parenchymal patterns: a meta-analysis of the published literature to examine the effect of method of classification. Cancer Detect Prev.

[44] Muhimmah I, Oliver A, Denton ER, Pont J, Pérez E, Zwiggelaar R (2006). Comparison between Wolfe, Boyd, BI-RADS and Tabár based mammographic risk assessment. Lect Notes Comput Sci..

[45] Gram IT, Bremnes Y, Ursin G, Maskarinec G, Bjurstam N, Lund E (2005). Percentage density, Wolfe's and Tabar's mammographic patterns: agreement and association with risk factors for breast cancer. Breast Cancer Res.

[46] Byng J, Boyd N, Fishell E, Jong R, Yaffe M (1996). Automated analysis of mammographic densities. Phys Med Biol.

[47] Caldwell CB, Stapleton SJ, Holdsworth DW, Jong RA, Weiser WJ, Cooke G (1990). Characterisation of mammographic parenchymal pattern by fractal dimension. Phys Med Biol.

[48] Magnin IE, Cluzeau F, Odet CL, Bremond A (1986). Mammographic texture analysis: an evaluation of risk for developing breast cancer. Opt Eng.

[49] Tahoces P, Correa J, Soutos M, Gomez L, Vidal J (1995). Computer-assisted diagnosis: the classification of mammographic breast parenchymal patterns. Phys Med Biol.

[50] Taylor P, Hajnal S, Dilhuydy M-H, Barreau B (1994). Measuring image texture to separate “difficult” from “easy” mammograms. Br J Radiol.

[51] Zheng Y, Keller BM, Ray S, Wang Y, Conant EF, Gee J (2015). Parenchymal texture analysis in digital mammography: a fully-automated pipeline for breast cancer risk assessment. Med Phys.

[52] Zheng Y, Wang Y, Keller BM, Conant E, Gee JC, Kontos D, editors. A fully-automated software pipeline for integrating breast density and parenchymal texture analysis for digital mammograms: parameter optimization in a case–control breast cancer risk assessment study. Orlando: SPIE Medical Imaging; International Society for Optics and Photonics; 2013.

[53] Sun W, Tseng T-LB, Qian W, Zhang J, Saltzstein EC, Zheng B (2015). Using multiscale texture and density features for near-term breast cancer risk analysis. Med Phys.

[54] Li H, Giger ML, Huo Z, Olopade OI, Lan L, Weber BL (2004). Computerized analysis of mammographic parenchymal patterns for assessing breast cancer risk: effect of ROI size and location. Med Phys.

[55] Huo Z, Giger ML, Olopade OI, Wolverton DE, Weber BL, Metz CE (2002). Computerized analysis of digitized mammograms of BRCA1 and BRCA2 gene mutation carriers. Radiology.

[56] Häberle L, Wagner F, Fasching PA, Jud SM, Heusinger K, Loehberg CR (2012). Characterizing mammographic images by using generic texture features. Breast Cancer Res.

[57] Haralick RM, Shanmugam K, Dinstein IH (1973). Textural features for image classification. Systems, Man and Cybernetics, IEEE Transactions on.

[58] Galloway MM (1975). Texture analysis using gray level run lengths. Comput Graph Image Process.

[59] Chu A, Sehgal CM, Greenleaf JF (1990). Use of gray value distribution of run lengths for texture analysis. Pattern Recogn Lett.

[60] Byng JW, Yaffe MJ, Lockwood GA, Little LE, Tritchler DL, Boyd NF (1997). Automated analysis of mammographic densities and breast carcinoma risk. Cancer.

[61] Nielsen M, Karemore G, Loog M, Raundahl J, Karssemeijer N, Otten JD (2011). A novel and automatic mammographic texture resemblance marker is an independent risk factor for breast cancer. Cancer Epidemiol.

[62] Reiser I, Lee S, Nishikawa RM (2011). On the orientation of mammographic structure. Med Phys.

[63] Choi JY, Ro YM (2012). Multiresolution local binary pattern texture analysis combined with variable selection for application to false-positive reduction in computer-aided detection of breast masses on mammograms. Phys Med Biol.

[64] Nielsen M, Vachon CM, Scott CG, Chernoff K, Karemore G, Karssemeijer N (2014). Mammographic texture resemblance generalizes as an independent risk factor for breast cancer. Breast Cancer Res..

[65] Ojala T, Pietikäinen M, Mäenpää T (2002). Multiresolution gray-scale and rotation invariant texture classification with local binary patterns. Pattern Anal Mach Intell, IEEE Trans..

[66] Manduca A, Carston MJ, Heine JJ, Scott CG, Pankratz VS, Brandt KR (2009). Texture features from mammographic images and risk of breast cancer. Cancer Epidemiol Biomarkers Prev.

[67] Zyout I, Czajkowska J, Grzegorzek M (2015). Multi-scale textural feature extraction and particle swarm optimization based model selection for false positive reduction in mammography. Comput Med Imaging Graph..

[68] Ford D, Easton DF, Bishop DT, Narod SA, Goldgar DE (1994). Risks of cancer in BRCA1-mutation carriers. Lancet.

[69] Mitchell G, Antoniou AC, Warren R, Peock S, Brown J, Davies R (2006). Mammographic density and breast cancer risk in BRCA1 and BRCA2 mutation carriers. Cancer Res.

[70] Gierach GL, Loud JT, Chow CK, Prindiville SA, Eng-Wong J, Soballe PW (2010). Mammographic density does not differ between unaffected BRCA1/2 mutation carriers and women at low-to-average risk of breast cancer. Breast Cancer Res Treat.

[71] Li H, Giger ML, Lan L, Janardanan J, Sennett CA (2014). Comparative analysis of image-based phenotypes of mammographic density and parenchymal patterns in distinguishing between BRCA1/2 cases, unilateral cancer cases, and controls. J Med Imaging.

[72] Torres-Mejia G, De Stavola B, Allen DS, Perez-Gavilan JJ, Ferreira JM, Fentiman IS (2005). Mammographic features and subsequent risk of breast cancer: a comparison of qualitative and quantitative evaluations in the Guernsey prospective studies. Cancer Epidemiol Biomarkers Prev.

[73] Wei J, Chan HP, Wu YT, Zhou C, Helvie MA, Tsodikov A (2011). Association of computerized mammographic parenchymal pattern measure with breast cancer risk: a pilot case–control study. Radiology.

[74] Brandt SS, Karemore G, Karssemeijer N, Nielsen M (2011). An anatomically oriented breast coordinate system for mammogram analysis. Med Imaging, IEEE Trans..

[75] Chen X, Moschidis E, Taylor C, Astley S. Breast cancer risk analysis based on a novel segmentation framework for digital mammograms. Medical Image Computing and Computer-Assisted Intervention–MICCAI 2014. Boston: Springer; 2014. p. 536–43. 10.1007/978-3-319-10404-1_67 25333160

[76] Wu Y-T, Sahiner B, Chan H-P, Wei J, Hadjiiski LM, Helvie MA, et al., editors. Comparison of mammographic parenchymal patterns of normal subjects and breast cancer patients. San Diego: Medical Imaging: International Society for Optics and Photonics; 2008

[77] Tan M, Qian W, Pu J, Liu H, Zheng B (2015). A new approach to develop computer-aided detection schemes of digital mammograms. Phys Med Biol.

[78] Tan M, Pu J, Cheng S, Liu H, Zheng B (2015). Assessment of a four-view mammographic image feature based fusion model to predict near-term breast cancer risk. Ann Biomed Eng.

[79] Keller BM, Chen J, Conant EF, Kontos D, editors. Breast density and parenchymal texture measures as potential risk factors for estrogen-receptor positive breast cancer. San Diego: SPIE Medical Imaging: International Society for Optics and Photonics; 2014 10.1117/12.2043710 PMC4112103 25075270

[80] Huo Z, Giger ML, Wolverton DE, Zhong W, Cumming S, Olopade OI (2000). Computerized analysis of mammographic parenchymal patterns for breast cancer risk assessment: feature selection. Med Phys.

[81] Li H, Giger ML, Olopade OI, Margolis A, Lan L, Chinander MR (2005). Computerized texture analysis of mammographic parenchymal patterns of digitized mammograms. Acad Radiol..

[82] Li H, Giger ML, Olopade OI, Lan L (2007). Fractal analysis of mammographic parenchymal patterns in breast cancer risk assessment. Acad Radiol.

[83] Li H, Giger ML, Olopade OI, Chinander MR. Power spectral analysis of mammographic parenchymal patterns for breast cancer risk assessment. J Digit Imaging. 2008;21(2):145–52. doi:10.1007/s10278-007-9093-9. 10.1007/s10278-007-9093-9 PMC3043857 18175183

[84] Li H, Giger ML, Lan L, Brown JB, MacMahon A, Mussman M (2012). Computerized analysis of mammographic parenchymal patterns on a large clinical dataset of full-field digital mammograms: robustness study with two high-risk datasets. J Digit Imaging.

[85] Gierach GL, Li H, Loud JT, Greene MH, Chow CK, Lan L (2014). Relationships between computer-extracted mammographic texture pattern features and BRCA1/2 mutation status: a cross-sectional study. Breast Cancer Res.

[86] Kontos D, Bakic PR, Carton AK, Troxel AB, Conant EF, Maidment ADA (2009). Parenchymal texture analysis in digital breast tomosynthesis for breast cancer risk estimation: a preliminary study. Acad Radiol.

[87] Kontos D, Ikejimba L, Bakic PR, Troxel AB, Conant EF, Maidment ADA (2011). Digital breast tomosynthesis parenchymal texture analysis: comparison with digital mammography and implications for cancer risk assessment. Radiology.

[88] Karemore G, Brand S, Sporring J, Nielsen M, editors. Anisotropic diffusion tensor applied to temporal mammograms: an application to breast cancer risk assessment. Engineering in Medicine and Biology Society (EMBC), 2010 Annual International Conference of the IEEE; 2010: IEEE. 10.1109/IEMBS.2010.5627183 21096598

[89] Karemore G, Nielsen M, Karssemeijer N, Brandt SS (2014). A method to determine the mammographic regions that show early changes due to the development of breast cancer. Phys Med Biol.

[90] Gastounioti A, Keller BM, Hsieh M-K, Conant EF, Kontos D, editors. Towards a breast-anatomy-weighted parenchymal texture signature for breast cancer risk assessment. Munich: Breast Image Analysis (BIA) Workshop, Medical Image Computing and Computer Assisted Intervention (MICCAI) Annual Meeting; 2015.

[91] Gastounioti A, Keller BM, Hsieh M-K, Conant EF, Kontos D, editors. Parenchymal texture measures weighted by breast anatomy: preliminary optimization in a case–control study. Munich: SPIE Medical Imaging: Computer-aided diagnosis; 2016.

[92] Gastounioti A, Oustimov A, Keller BM, Pantalone L, Hsieh M-K, Conant EF, et al., editors. Associations of dense and fatty breast-tissue heterogeneity with breast cancer risk: Preliminary evaluation using parenchymal texture measurements driven by breast anatomy. San Diego: Radiological Society of North America (RSNA) Annual Meeting; 2015.

[93] LeCun Y, Bengio Y, Hinton G (2015). Deep learning. Nature.

[94] Kallenberg M, Petersen K, Nielsen M, Ng A, Diao P, Igel C (2016). Unsupervised deep learning applied to breast density segmentation and mammographic risk scoring. IEEE Trans Med Imaging.

[95] Petersen K, Nielsen M, Diao P, Karssemeijer N, Lillholm M. Breast tissue segmentation and mammographic risk scoring using deep learning. Breast Imaging: Springer; 2014. p. 88–94

[96] Qiu Y, Wang Y, Yan S, Tan M, Cheng S, Liu H, et al., editors. An initial investigation on developing a new method to predict short-term breast cancer risk based on deep learning technology. Gifu City: SPIE Medical Imaging: Computer-aided diagnosis; 2016

[97] Friedewald SM, Rafferty EA, Rose SL, Durand MA, Plecha DM, Greenberg JS (2014). Breast cancer screening using tomosynthesis in combination with digital mammography. JAMA.

[98] Sechopoulos I (2013). A review of breast tomosynthesis. Part II. Image reconstruction, processing and analysis, and advanced applications. Med Phys.

[99] Sechopoulos I (2013). A review of breast tomosynthesis. Part I. The image acquisition process. Med Phys.

[100] Houssami N, Miglioretti DL (2016). Digital breast tomosynthesis: a brave new world of mammography screening. JAMA Oncol.

[101] Keller BM, Oustimov A, Wang Y, Chen J, Acciavatti RJ, Zheng Y (2015). Parenchymal texture analysis in digital mammography: robust texture feature identification and equivalence across devices. J Med Imaging.

[102] Heine JJ, Malhotra P (2002). Mammographic tissue, breast cancer risk, serial image analysis, and digital mammography. Part 1. Tissue and related risk factors. Acad Radiol.

[103] Heine JJ, Malhotra P (2002). Mammographic tissue, breast cancer risk, serial image analysis, and digital mammography: Part 2. Serial breast tissue change and related temporal influences. Acad Radiol.

[104] Li H, Giger ML, Sun C, Ponsukcharoen U, Huo D, Lan L (2014). Pilot study demonstrating potential association between breast cancer image-based risk phenotypes and genomic biomarkers. Med Phys.

[105] Russo J, Lynch H, Russo IH (2001). Mammary gland architecture as a determining factor in the susceptibility of the human breast to cancer. Breast J.

[106] Lambin P, Rios-Velazquez E, Leijenaar R, Carvalho S, van Stiphout RG, Granton P (2012). Radiomics: extracting more information from medical images using advanced feature analysis. Eur J Cancer.

[107] Kuo MD, Jamshidi N (2014). Behind the numbers: decoding molecular phenotypes with radiogenomics—guiding principles and technical considerations. Radiology.

[108] Yamamoto S, Maki DD, Korn RL, Kuo MD (2012). Radiogenomic analysis of breast cancer using MRI: a preliminary study to define the landscape. Am J Roentgenol.

[109] Mazurowski MA, Zhang J, Grimm LJ, Yoon SC, Silber JI (2014). Radiogenomic analysis of breast cancer: luminal B molecular subtype is associated with enhancement dynamics at MR imaging. Radiology.

[110] Mendel KR, Li H, Giger ML, editors. Quantitative breast MRI radiomics for cancer risk assessment and the monitoring of high-risk populations. San Diego: SPIE Medical Imaging: International Society for Optics and Photonics; 2016

[111] Guo W, Li H, Zhu Y, Lan L, Yang S, Drukker K (2015). Prediction of clinical phenotypes in invasive breast carcinomas from the integration of radiomics and genomics data. J Med Imaging.

